# DO INTERNALIZED AGE STEREOTYPES MEDIATE THE RELATIONSHIP BETWEEN VOLUNTEERING AND SELF-EFFICACY AMONG ADULTS 50+?

**DOI:** 10.1093/geroni/igac059.431

**Published:** 2022-12-20

**Authors:** Andrew Steward, Leslie Hasche

**Affiliations:** University of Wisconsin-Milwaukee, Milwaukee, Wisconsin, United States; University of Denver, Denver, Colorado, United States

## Abstract

Volunteering is associated with increased self-efficacy among older adults, and self-efficacy has been shown to mediate the relationship between ageism and health. A growing, compelling body of literature based in stereotype embodiment theory identifies significant, negative public health impacts of internalized age stereotypes. Yet, little research explores whether volunteering may both reduce internalized ageism and enhance self-efficacy as people age. This cross-sectional study examined whether internalized age stereotypes mediate the relationship between volunteering and self-efficacy for adults 50+. A convenience sample of volunteers (n = 165) 50+ years of age residing in the U.S. Mountain West was recruited. A 15-minute, online survey was utilized. The independent variable was number of volunteer hours per week (mean = 6.45, SD = 5.38). The dependent variable was self-efficacy measured by five items from the five-point, Likert-type general self-efficacy scale (α = .83; mean = 4.81, SD = 1.08). Drawing from the self-stereotypes of aging scale, the indirect effects of five internalized positive (e.g., “wise” and “capable”) and five negative (e.g., “grumpy” and “helpless”) age stereotypes were tested. Results indicate that increased internalized positive, but not negative, age stereotypes partially mediated the relationship between volunteer hours and self-efficacy while holding constant age, gender, race, self-rated health, functional limitation, education, employment, and previous volunteer experience. Although positive age stereotypes have long been considered a form of ageism, the results of this study suggest that internalized positive age stereotypes may function as a form of esteem to promote enhanced psychosocial health as people age.

